# Diversity Characteristics and Composition of Gut Microbiota in *Antheraea pernyi* (Lepidoptera: Saturniidae) Larvae Across Different Instars

**DOI:** 10.3390/insects16090909

**Published:** 2025-09-01

**Authors:** Peng Hou, Li Liu, Xin Ma, Ying Men, Ding Yang, Jianfeng Wang, Chuntian Zhang

**Affiliations:** 1 College of Life Science and Engineering, Shenyang University, Shenyang 110044, China; hp_0829@syu.edu.cn (P.H.); liujiali0229@163.com (L.L.); maxin20001105@163.com (X.M.); menying1105@163.com (Y.M.); 2 College of Plant Protection, China Agricultural University, Beijing 110193, China; dyangcau@126.com; 3 College of Life Science, Shenyang Normal University, Shenyang 110034, China

**Keywords:** gut microbiota, *Antheraea pernyi* larvae, different instars, lepidopteran insects, 16S rRNA

## Abstract

*Antheraea pernyi* (Lepidoptera: Saturniidae) is an economically important silk-producing insect, whose gut microbiota play a crucial role in growth, development, and nutrient metabolism. This study focused on the entire larval developmental stages of *A. pernyi.* Using the Illumina MiSeq high-throughput sequencing platform, we performed 16S rRNA gene amplicon sequencing on the gut microbiota of laboratory-reared *A. pernyi* larvae, analyzing in detail the composition and diversity characteristics of the gut microbial communities across all five instars (1st to 5th instar). Additionally, functional predictions were conducted to explore the potential roles of these microbiota during larvae development. The study revealed that the core gut microbiota of *A. pernyi* larvae primarily consisted of Actinomycetota (39.78%), Cyanobacteriota (32.46%), Bacillota (18.08%), and Pseudomonadota (9.02%). Among these, Actinomycetota dominated in the 1st to 4th-instar larvae, while Cyanobacteriota became the predominant phylum in the 5th instar. Linear discriminant analysis effect size identified statistically significant biomarkers across different instar larvae of *A. pernyi.* Alpha diversity analysis showed that gut microbiota diversity initially increased and then decreased with larval development, peaking in the 3rd instar, and reaching its lowest level in the 5th instar. Principal coordinate analysis (PCoA) of beta diversity indicated that the gut microbiota structures of the 1st to 4th instars were similar but significantly differed from that of the 5th instar. Functional prediction analysis based on the KEGG database revealed that Carbohydrate metabolism and Amino acid metabolism-related genes were significantly lower in the 5th instar compared to other instars, while Energy metabolism and Cofactor and vitamin metabolism-related genes were significantly higher. This study offers valuable insights for the development of gut microbial resources in Lepidoptera insects.

## Simple Summary

Gut microbiota profoundly influence the biological functions and ecological adaptability of host insects. This study conducted in-depth research on the gut microbiota across different larval stages (1st to 5th instar) of the important resource insect *Antheraea pernyi* (laboratory-reared), revealing its composition, diversity characteristics, and potential functions. These findings enrich the gut microbiota resource library of lepidopteran insects, establish a crucial foundation for understanding host-microbe interactions in this group, and provide key insights for microbial resource utilization and risk assessment in the development of the Tussah silk industry.

## 1. Introduction

*Antheraea pernyi* (Chinese oak silkworm), belonging to the genus *Antheraea* within the order Lepidoptera (family Saturniidae), is a silk-producing insect resource [1,2]. China is the origin of this species and has maintained its position as the world’s leading producer of oak silkworm cocoons for over 2000 years, accounting for 90% of global production [3,4]. As a high value-added agricultural product, *A. pernyi* is extensively farmed in Chinese provinces and regions such as Liaoning, Jilin, Inner Mongolia, Heilongjiang, and Henan, representing immense economic value. With social development and scientific advancement, the utilization of oak silkworms extends beyond traditional applications like food and silk textiles, revealing significant potential in fields such as healthcare, biopesticides, and novel materials [1,3,5,6].

As a holometabolous insect, the development of *A. pernyi* progresses through four distinct stages: egg, larva, pupa, and adult. Among these, the larval stage represents the critical period for nutrient accumulation and growth development [7,8]. The larvae of *A. pernyi* are nutritionally rich, containing abundant unsaturated fatty acids and high-quality proteins. Existing research has confirmed that their protein content accounts for 62.3%, while the abundant alpha-linolenic acid constitutes 45.3% of the total fatty acids—a concentration more than 50 times higher than that found in common animal foods [4]. As a phytophagous insect that primarily feeds on *Quercus mongolica* leaves, the larvae ‘s feeding habits make it vulnerable to potential pathogens in the environment, leading to reduced yields and economic losses [8]. Consequently, scientific and technological discussions focusing on healthy rearing and biological control of *A. pernyi* have garnered sustained attention from numerous scholars, with the interaction mechanisms between insects and gut microbiota representing a significant perspective.

The larvae of Lepidoptera exhibit a segmented body structure, with a head bearing sensory organs and mandibles, three pairs of true legs on the thorax, and four pairs of prolegs on the abdomen [9]. Their digestive tract is relatively simple and tubular, comprising the foregut, midgut, and hindgut [10,11]. The insect gut harbors diverse microbial communities including bacteria, fungi, archaea, and viruses, with bacteria being the most abundant constituents [12]. With long-term coevolution, these gut microorganisms have developed diverse population structures and biological functions [13]. Research indicates significant variations in gut microbial composition among different lepidopteran species, driven by factors such as host developmental stage, growth environment, and food sources, which play crucial roles in host health and adaptation [11]. For instance, they assist hosts in degrading macromolecules like plant cellulose, provide essential amino acids and vitamins unavailable through host biosynthesis, and participate in detoxification metabolism and immune defense [10,14]. With rapid advances in modern molecular biology, studies on dynamic changes in gut microbiota during lepidopteran development have increased substantially. Research has revealed declining gut microbial diversity in *Tuta absoluta* larvae as they progress through instars [15]. Significant alterations in gut microbiota structure and diversity occur across different developmental stages of *Spodoptera frugiperda* [16].

To date, research on the gut microbiota of the *A. pernyi* has primarily focused on analyzing the composition and diversity. This work provides an important reference for studying the insect’s stress resistance, developing microbial resources, and assessing risks to the development of the oak silkworm industry. Zou et al. isolated six bacterial species belonging to three genera (*Bacillus*, *Staphylococcus*, and *Enterobacter*) from the midguts of healthy 5th-instar larvae [17]. He et al., utilizing high-throughput SOLEXA sequencing, detected 87 bacterial species across 37 genera in diseased pupae [18]. Zhang et al. comparatively analyzed the gut microbiota composition of midgut tissues and midgut contents in 5th-instar larvae using 16S rRNA gene sequencing, identifying 71 genera from 10 phyla [19]. Xu et al. compared the gut bacterial diversity across different days within the 5th-instar larval stage, annotating 196 genera and 235 species [7]. However, existing studies are limited by experimental materials and practical constraints, resulting in a lack of systematic investigation into the gut microbiota of *A. pernyi* larvae. Few studies have examined the microbiota across the insect’s entire developmental process through all five instars, and comparative analyses of microbiota composition and diversity across these larval stages are scarce. This gap hinders a comprehensive understanding of the *A. pernyi*-microbe symbiotic mechanisms. Therefore, this study employed laboratory-reared 1st to 5th-instar larvae as experimental material, utilized high-throughput sequencing of the 16S rRNA gene, analyzing the composition and diversity characteristics of their gut microbiota, and combined this with functional prediction to explore the potential roles. This study aims to enrich the resource library of insect gut microbiota while providing a reference for research on gut microbiota in Lepidoptera insects.

## 2. Materials and Methods

### 2.1. Laboratory Rearing of A. pernyi Larvae

The experimental *A. pernyi* were derived from the ‘Ji Qing’ strain, selectively bred by the Jilin Provincial Academy of Sericultural Sciences. This strain underwent 5 years (10 generations) of systematic selection followed by 6 years (12 generations) of mass selection, resulting in excellent high-yield performance and strong hybrid combining ability. Under natural conditions, it completes two generations per year.

**Figure 1:**
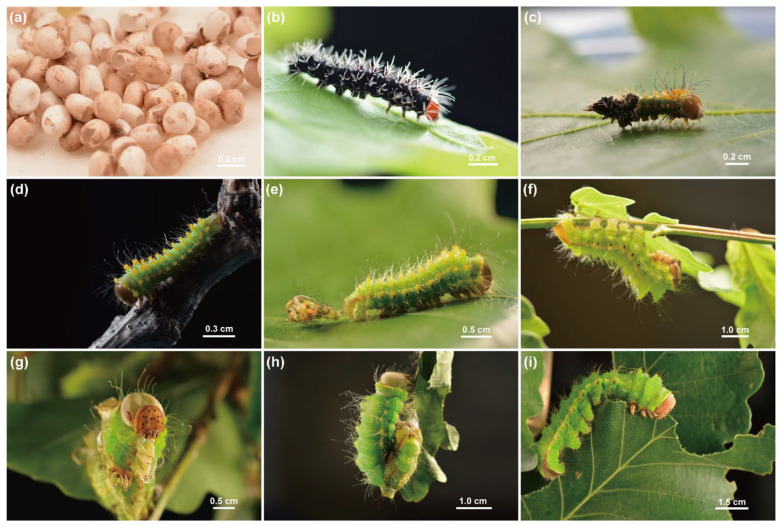
Morphology of *A. pernyi* larvae across various instars. (**a**) Eggs; (**b**) 1st-instar larva; (**c**) the molting process from 1st-instar larva to 2nd-instar larva; (**d**) 2nd-instar larva; (**e**) the molting process from 2nd-instar larva to 3rd-instar larva; (**f**) 4th-instar larva; (**g**,**h**) the molting process from 4th-instar larva to 5th-instar larva; (**i**) 5th-instar larva. Photo by Peng Hou.

The morphological characteristics are as follows (Figure 1): Eggs were brown with white chorions and elliptical in shape, newly hatched (1st-instar) larvae possessed reddish-brown heads and dark black bodies. From the 2nd to 5th instar, the larval body surface was bluish green, with 5th-instar larvae specifically showing bud green dorsally and parrot green laterally. The larval development encompassed five instars, undergoing four molts.

The larvae were reared under controlled conditions at the Liaoning Provincial Key Laboratory of Urban Pest Management and Ecological Security, starting from eggs hatched in April 2025. The total rearing duration to the end of the 5th instar was 48 ± 2 days. Larvae were maintained in a 16 h light: 8 h dark photoperiod and fed daily surface-cleaned fresh leaves of healthy *Quercus mongolica*. Environmental parameters were staged: 1st–2nd instars (6–8 days) at 20 ± 2 °C and 85% RH; 3rd instars (6–8 days) at 22 ± 2 °C and 80 ± 5% RH; 4th instars (11–13 days) and 5th instars (18–20 days) both at 22 ± 2 °C and 70 ± 5% RH.

### 2.2. Extraction of Microbiota Total DNA from the Gut of A. pernyi Larvae

During laboratory rearing, 30 healthy larvae were randomly selected from each instar (1st, 2nd, 3rd, 4th, and 5th instar), with all larvae being 2 days into their respective developmental stages. Three replicates were set up for each instar group to meet statistical standards. The selected *A. pernyi* larvae were starved for 24 h at room temperature, then placed in a biosafety cabinet (BSC) and surface-sterilized with 75% ethanol three times (60 s each), followed by three rinses with sterile water. The cleaned larvae were dissected in sterile water to obtain gut samples, which were then washed three times in 0.9% NaCl solution, transferred to 1.5 mL centrifuge tubes, flash-frozen in liquid nitrogen, and stored at −80 °C for further use. Ultimately, 15 gut sample groups (5 instars × 3 replicates each) were obtained.

### 2.3. 16S rRNA Amplification and Sequencing of Gut Microbiota

The gut tissue samples of *A. pernyi* larvae were homogenized using a vortex mixer, and total DNA of gut microbiota was extracted strictly following the manufacturer’s instructions of the E.Z.N.A.^®^ Soil DNA Kit (Omega Bio-tek, Norcross, GA, USA). DNA integrity was assessed via 1% agarose gel electrophoresis, and the V3-V4 hypervariable regions of the bacterial 16S rRNA gene were amplified using universal primers 338F (5′-ACTCCTACGGGAGGCAGCAG-3′) and 806R (5′-GGACTACHVGGGTWTCTAAT-3′). The PCR reaction mixture (20 μL) comprised AB 2×MIX (20 μL), forward primer (5 μM, 0.8 μL), reverse primer (5 μM, 0.8 μL), template DNA (10 ng/μL), and ddH_2_O to adjust the final volume. To ensure experimental reliability, a negative control was incorporated into the PCR system by substituting template DNA with ddH_2_O, while maintaining identical concentrations of all other reaction components compared to the experimental group. Amplification conditions included initial denaturation at 95 °C for 3 min, followed by 29 cycles of denaturation (95 °C, 30 s), annealing (55 °C, 30 s), and extension (72 °C, 45 s), with a final extension at 72 °C for 10 min and holding at 4 °C (Thermal cycler: ABI GeneAmp^®^ 9700, Union City, CA, USA). PCR products were separated via 2% agarose gel electrophoresis, and target bands were purified using the AxyPrep DNA Gel Extraction Kit (Axygen, Union City, CA, USA). Purified amplicons were quantified with a Qubit 4.0 Fluorometer (Thermo Fisher Scientific, Waltham, MA, USA). Sequencing libraries were constructed using the NEXTFLEX Rapid DNA-Seq Kit, and paired-end sequencing (PE 300) was conducted on the Illumina MiSeq platform by Shanghai Majorbio Bio-Pharm Technology Co., Ltd. (Shanghai, China).

### 2.4. Processing of Sequencing Data from the Gut of A. pernyi Larvae

The paired-end raw sequencing reads were quality-controlled using fastp (https://github.com/OpenGene/fastp accessed on 29 June 2025, version 0.23.4) and assembled using FLASH (https://ccb.jhu.edu/software/FLASH/index.shtml accessed on 30 June 2025, version 1.2.11). Specifically, the following steps were performed: First, bases with a quality score below 20 at the tail of the reads were filtered. A sliding window of 50 bp was applied, and if the average quality within the window was below 20, the trailing bases starting from the window were trimmed. Reads shorter than 50 bp after quality control were discarded and reads containing N bases were removed. Second, paired-end reads were merged into a single sequence based on their overlap, with a minimum overlap length of 10 bp. Third, the maximum mismatch ratio allowed in the overlapping region was set to 0.2, and sequences failing to meet this criterion were filtered out. Finally, samples were distinguished based on the barcode and primer sequences at both ends of the sequences, and the orientation of the sequences was adjusted (with zero mismatches allowed in the barcode and a maximum of two mismatches allowed in the primer).

Using the DADA2 plugin (https://qiime2.org accessed on 1 July 2025, version 2024) in the QIIME2 platform, the optimized sequences after quality control and assembly were denoised to obtain amplicon sequence variants (ASVs) [20]. To minimize the impact of sequencing depth on subsequent alpha and beta diversity analyses, all optimized sequence counts were rarefied to the minimum data volume among the samples (34,520 sequences per sample). After rarefaction, the average sequence coverage of each sample still reached 99.09%. Based on the SILVA 16S rRNA gene database (https://www.arb-silva.de/ accessed on 2 July 2025, version 138), taxonomic classification of ASVs was performed using the Naive Bayes classifier in QIIME2. Functional prediction of 16S rRNA was conducted using PICRUSt2 (version 2.2.0) (https://github.com/picrust/picrust2/ accessed on 3 July 2025).

### 2.5. Statistical Analysis of Sequencing Data from the Gut of A. pernyi Larvae

All data Statistical analyses were completed on the Majorbio Bioinformatics Cloud Platform (https://cloud.majorbio.com accessed on 4 July 2025). The specific steps were as follows: Species rarefaction curves were generated using ASV data to validate data reliability. The MOTHUR software (http://www.mothur.org/wiki/Calculators accessed on 4 July 2025, version 1.30.2) was used to calculate species diversity indices, and the Kruskal–Wallis rank-sum test was employed for intergroup differences analysis of Alpha diversity. Based on the Bray–Curtis distance algorithm, the similarity of microbiota structures between samples was examined, and principal coordinate analysis (PCoA) was performed to visualize the relationship between overall and sample groupings, analyzing whether the differences in microbiota structures between sample groups were significant. Linear discriminant analysis effect size (LefSe) (http://huttenhower.sph.harvard.edu/LEfSe accessed on 5 July 2025) was employed to identify statistically significant biomarkers (LDA score > 4) among different instars, revealing the most discriminative taxonomic features that likely explain the differences in microbiota. Finally, metabolic functional prediction analysis of microbial communities was conducted based on KEGG gene function annotation.

## 3. Results

### 3.1. General Sequencing Data Results of Gut Microbiota in A. pernyi Larvae

**Figure 2:**
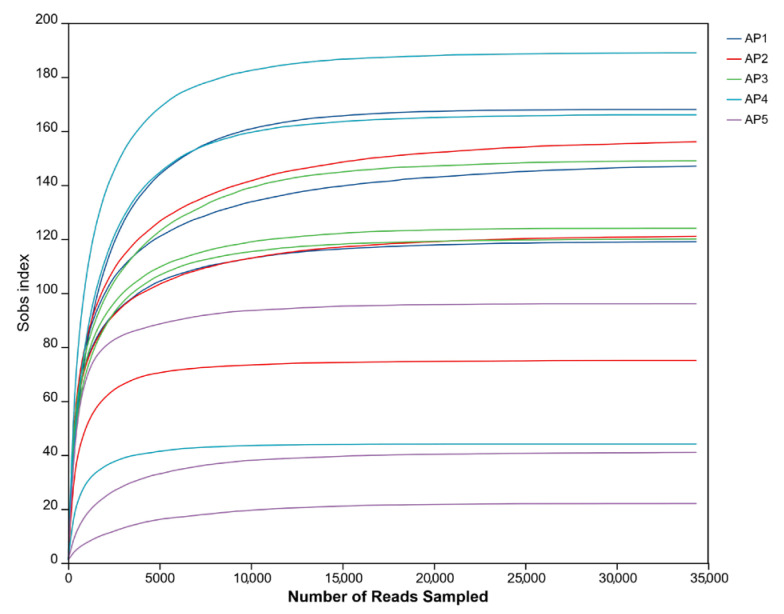
Rarefaction curve of microbiota in the gut of *A. pernyi* larvae across different instars.

Using the Illumina MiSeq platform, we conducted paired-end sequencing of the V3-V4 hypervariable regions of 16S rRNA gene amplicons derived from 15 samples (3 replicates for each instar from AP1 to AP5). After quality control and optimization, a total of 923,206 high-quality sequences were obtained, with an average length of 413 bp (Table 1). Denoising was performed using DADA2, which involved comparing single-nucleotide differences and clustering quality-filtered sequences. This process yielded a total of 789 amplicon sequence variants (ASVs) across all samples, with a minimum of 23 in sample AP5_1 and a maximum of 189 in AP4_2. Among these, the average number of ASVs in 1st-instar larvae (AP1) was significantly higher than in other developmental instars. Rarefaction curve analysis showed that as sequencing depth increased, the number of observed species in each sample group gradually plateaued and eventually reached saturation (Figure 2). These results demonstrate the reliability of the experimental data, accurately reflecting the true composition of microbial communities in the samples.

### 3.2. Composition and Differences In The Gut Microbiota in A. pernyi Larvae

**Figure 3:**
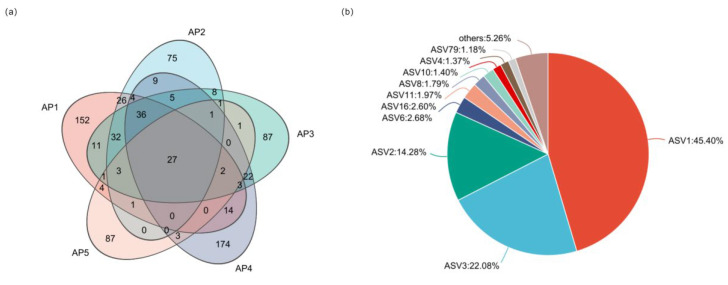
Venn diagram and pie chart of the ASVs in *A. pernyi* larvae across different instars. (**a**) Venn diagram of the ASVs in *A. pernyi* larvae across different instars. (**b**) Pie chart of shared 27 ASVs across all instar stages.

According to the visualized Venn diagram of microbiota ASVs in *A. pernyi* larvae across different instars (Figure 3a), the unique ASV counts for 1st, 2nd, 3rd, 4th, and 5th-instar larvae were 152, 75, 87, 174, and 87, respectively, accounting for 19.26%, 9.51%, 11.03%, 22.05%, and 11.03% of the total microbiota ASVs. The number of shared ASVs across all instar stages was 27, representing 3.42% of the total (Figure 3b). Herein, ASV1 is *unclassified_g__norank_o__Chloroplast*, ASV2 is Nitriliruptoraceae, ASV3 is *Dietzia*, ASV8 is *Halomonas*, ASV16 is *Nesterenkonia*_sp._MY13, along with 17 other ASV taxonomic units.

**Figure 4:**
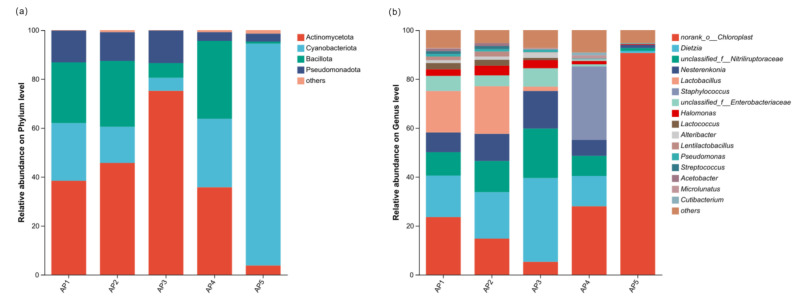
Composition of gut microbiota of *A. pernyi* larvae across different instars. (**a**) At the phylum level. (**b**) At the genus level. Note: The horizontal axis represents instars 1st, 2nd, 3rd, 4th, and 5th; the vertical axis represents the relative microbiota abundances in the sample.

Taxonomic analysis of sequencing data revealed that at the phylum level, the core microbiota in the larval gut of *A. pernyi* primarily consisted of Actinomycetota, Cyanobacteriota, Bacillota, and Pseudomonadota. Among these, Actinomycetota was the dominant phylum in the 1st, 2nd, 3rd, and 4th-instar larvae, accounting for 38.41%, 45.72%, 75.20%, and 35.77%, respectively, while Cyanobacteriota became the dominant phylum in the 5th-instar larvae, representing 90.67% (Figure 4a). At the genus level, the core gut microbiota mainly included *Dietzia*, *unclassified_f__Nitriliruptoraceae*, *Nesterenkonia*, and *Lactobacillus*. Specifically, *Dietzia* was the dominant genus in the 2nd and 3rd-instar larvae (19.00% and 34.24%). The 4th-instar larvae were primarily colonized by *Staphylococcus* (30.05%) (Figure 4b).

**Figure 5:**
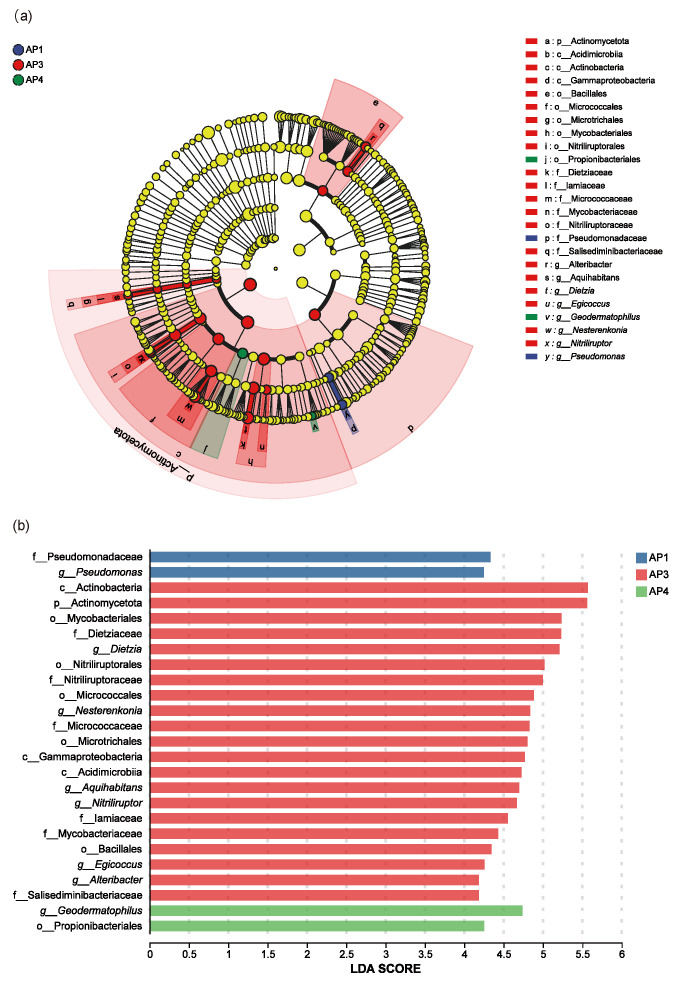
LEfSe analysis of gut microbiota of *A. pernyi* larvae across different instars. (**a**) Cladogram of microbial taxa enriched across different instars. Distinct colors indicate microbial communities significantly enriched in a specific group and contributing to inter-group differences; yellow indicates non-significant communities. (**b**) LDA discriminates histogram. Note: Phyla (p), Class (c), Order (o), Family (f), Genus (g).

Linear discriminant analysis effect size (LefSe) was employed to identify microbial taxa that significantly characterize the differences in gut microbiota across developmental instars (Linear discriminant analysis, LDA > 4). The results revealed significantly enriched microbial taxa in the guts of 1st-, 3rd-, and 4th-instar *A. pernyi* larvae, with 25 microbial taxonomic units showing significant differences identified (Figure 5a).

Specifically, at the phylum level, Actinobacteria was significantly enriched in 3rd-instar larvae. At the class level, Acidimicrobiia, Actinobacteria, and Gammaproteobacteria were significantly enriched in 3rd-instar larvae. At the order level, Propionibacteriales was significantly enriched in 4th-instar larvae, while Bacillales, Micrococcales, Microtrichales, Mycobacteriales, and Nitriliruptorales were all significantly enriched in 3rd-instar larvae. At the family level, Pseudomonadaceae was significantly enriched in 1st-instar larvae, while Dietziaceae, Iamiaceae, Micrococcaceae, Mycobacteriaceae, Nitriliruptoraceae, and Salisediminibacteriaceae were all significantly enriched in 3rd-instar larvae. At the genus level, *Geodermatophilus* was significantly enriched in 4th-instar larvae, while *Alteribacter, Aquihabitans, Dietzia, Egioccus, Nesterenkonia, Nitriliruptor*, and *Pseudomonas* were significantly enriched in 3rd-instar larvae. (Figure 5b).

**Figure 6:**
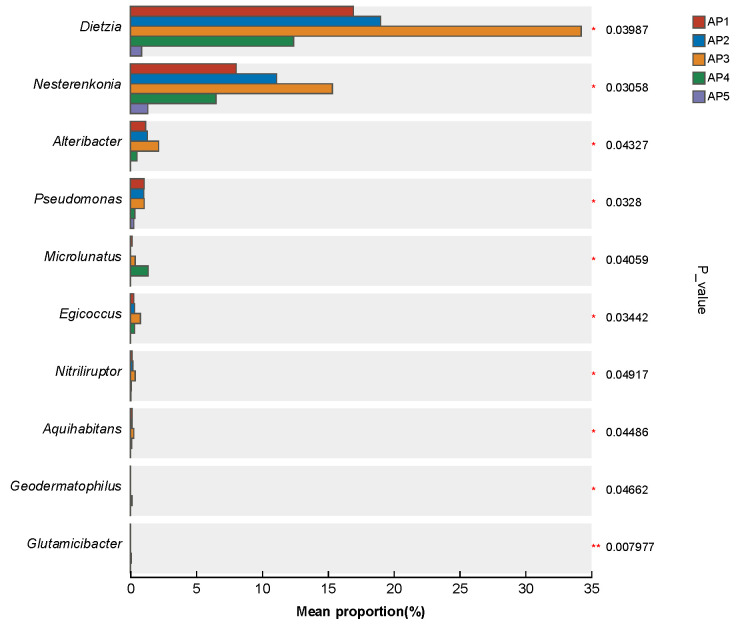
Significance test of genus differences in the gut microbiota of *A. pernyi* larvae. Note: * 0.01 < *p* ≤ 0.05, ** 0.001 < *p* ≤ 0.01.

Through significance testing of interspecies differences in the gut microbiota of *A.pernyi* larvae, it was found that the abundance of gut microbiota dynamic changes with larval growth. The contents of *Dietzia, Nesterenkonia, Alteribacter*, and *Pseudomonas* in the larval gut varied markedly from the 1st to the 5th instar, with their abundances gradually increasing from the 1st to the 3rd instar, peaking in the 3rd instar, declining in the 4th instar, and reaching the lowest level in the 5th instar (Figure 6).

**Figure 7:**
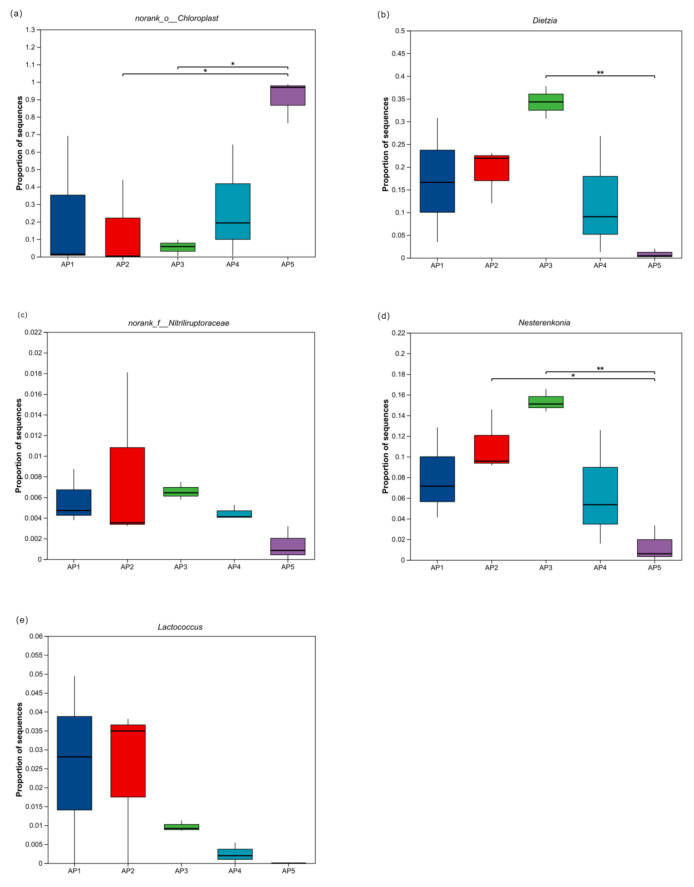
Analysis of compositional differences in the gut microbiota of *A. pernyi* larvae across different instars. (**a**) *norank_o__Chloroplast.* (**b**) *Dietzia.* (**c**) *Nesterenkonia*. (**d**) *unclassified_f__Nitriliruptoraceae.* (**e**) *Lactobacillus.* Note: * 0.01 < *p* ≤ 0.05, ** 0.001 < *p* ≤ 0.01.

Further analysis of the compositional differences in the core gut microbiota of larvae across different developmental instars revealed that the abundance of *norank_o__Chloroplast* in the gut of 5th-instar was significantly higher than that in 2nd- and 3rd-instar larvae (*p* < 0.05). The abundances of *Dietzia* and *Nesterenkonia* in the gut of 3rd-instar larvae were significantly higher than those in 5th-instar larvae (*p* < 0.01). Additionally, the abundance of *unclassified_f__Nitriliruptoraceae* in 3rd-instar larvae was significantly higher than that in 4th-instar larvae (*p* < 0.01) and markedly higher than that in 5th-instar larvae (*p* < 0.05). In contrast, no significant differences were observed in the abundance of *Lactobacillus* across different larval stages (Figure 7).

### 3.3. Diversity Characteristics of the Gut Microbiota in A. pernyi Larvae

#### 3.3.1. Alpha Diversity Analysis

The Alpha diversity indices in *A. pernyi* larvae at different developmental instars indicate that the diversity of gut microbiota initially increases and then decreases with larval growth. The gut microbiota diversity is highest in 3rd-instar larvae, as evidenced by the highest Shannon index and the lowest Simpson index. In contrast, the 5th-instar larvae exhibit the lowest microbiota diversity, with the lowest Shannon index and the highest Simpson index. Additionally, the richness of the gut microbiota shows a declining trend as larval development progress. Specifically, the ACE and Chao indices are highest in 1st-instar larvae (AP1), fluctuate considerably in 2nd- to 4th-instar larvae (AP2–AP4), and significantly decrease to the lowest levels in 5th-instar larvae (AP5) (Table 2).

**Figure 8:**
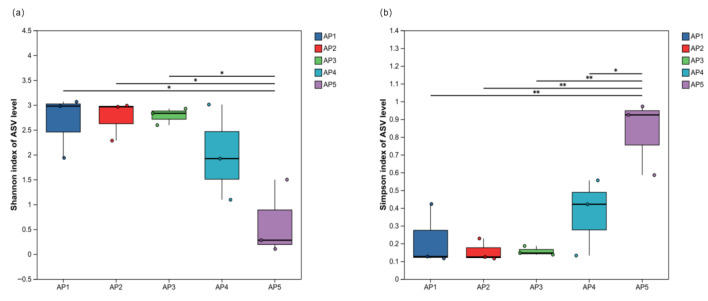
Significance tests for intergroup differences in gut microbiota diversity indices of *A. pernyi* larvae across different instars. (**a**) Shannon index of *A. pernyi* larvae across different instars; (**b**) Simpson index of *A. pernyi* larvae across different instars. Note: The asterisk represented significant differences. *: *p* ≤ 0.05, **: *p* ≤ 0.01.

The intergroup difference test of the Alpha diversity index revealed significant variations in the diversity of gut microbiota across different instar larvae of the *A. pernyi.* The Shannon index of 5th-instar larvae was significantly lower than that of 1st, 2nd, and 3rd-instar larvae (*p* < 0.05), but showed no significant difference compared to 4th-instar larvae. The Simpson index of 5th-instar larvae was significantly higher than that of 1st, 2nd, and 3rd-instar larvae (*p* < 0.01) and significantly higher than that of 4th-instar larvae (*p* < 0.05) (Figure 8). These results indicate that the microbiota diversity in the gut of 5th-instar larvae is significantly lower than that of 1st to 4th-instar larvae.

#### 3.3.2. Beta Diversity Analysis

The results of principal co-ordinates analysis (PCoA) based on ASV annotation level revealed significant differences in the gut microbiota structure of *A. pernyi* larvae across different developmental instars. The contribution rates of PCoA1 and PCoA2 to sample variation were 62.16% and 20.03%, respectively, with a cumulative contribution rate of 80.19%, reflecting 80.19% of the total inter-sample differences. Samples from the AP1, AP2, AP3, and AP4 groups were tightly clustered in spatial distribution, while the AP5 group was distinctly separated from the other groups. This indicates that the gut microbiota structure of 1st-, 2nd-, 3rd-, and 4th-instar larvae was similar but significantly different from that of 5th-instar larvae.

**Figure 9:**
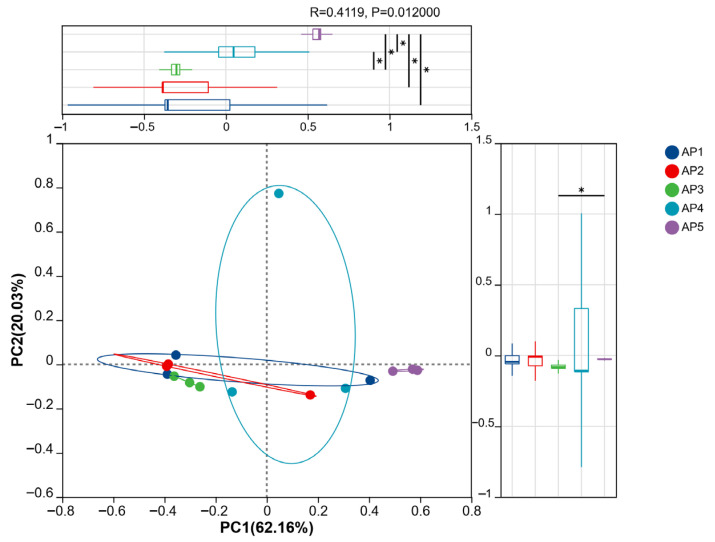
PCoA was performed on the ASV classification levels of gut microbiota in *A. pernyi* larvae. Note: * 0.01 < *p* ≤ 0.05.

Additionally, the distances between samples represent the differences in microbiota structure, with closer distances indicating greater similarity in microbiota composition and smaller differences. The AP5 group exhibited the smallest and most uniform spatial distances between samples, reflecting the least inter-individual variation in microbiota structure. The AP3 group showed moderate variation, while the AP1, AP2, and AP4 groups displayed high dispersion of sample points, indicating greater variability in gut microbiota composition among individuals within these groups (Figure 9).

### 3.4. Functional Prediction of Gut Microbiota in A. pernyi Larvae Across Different Instars

**Figure 10:**
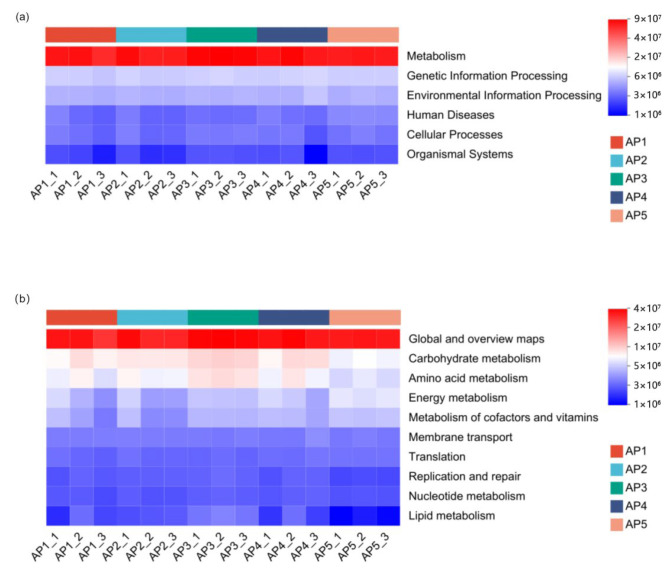
Annotations of the KEGG pathways for gut microbiota gene functions. (**a**) KEGG primary pathway annotation level. (**b**) KEGG secondary pathway annotation level.

**Figure 11:**
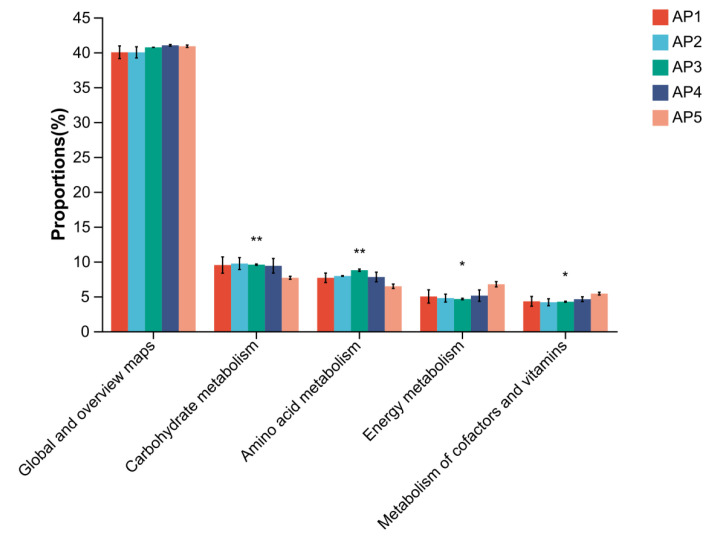
Analysis of gene functional differences in the gut microbiota of *A. pernyi* larvae. *: *p* ≤ 0.05, **: *p* ≤ 0.01.

The gene function prediction analysis based on the KEGG database revealed that the primary metabolic pathways annotated in the gut microbiota of *A. pernyi* larvae across different instars included (Figure 10): Metabolism, Genetic Information Processing, Environmental Information Processing, Human Diseases, Cellular Processes, and Organismal Systems. Among these, the Metabolism accounted for the highest proportion, followed by Genetic Information Processing and Environmental Information Processing. The following secondary metabolic pathways included: Carbohydrate metabolism, Amino acid metabolism, Energy metabolism, Metabolism of cofactors and vitamins, Nucleotide metabolism, and Lipid metabolism. Additionally, it included Translation and Membrane transport, as well as fundamental functions such as Global and overview maps, and Replication and repair. Further analysis of the functional differences in microbiota genes revealed that the functional genes related to Global and overview maps showed no significant differences across various instars of larvae. However, the functional genes associated with Carbohydrate metabolism and Amino acid metabolism in 5th-instar larvae were significantly lower than those in other instars, while the genes related to Energy metabolism and Metabolism of cofactors and vitamins in 5th-instar larvae were significantly higher than those in other instars (Figure 11).

## 4. Discussion

Reported studies have confirmed that at the phylum level, the gut microbiota of lepidopteran insects is predominantly composed of Proteobacteria, Firmicutes, and Actinobacteria [21]. In this study, we identified Actinomycetota (39.78%), Cyanobacteriota (32.46%), Bacillota (formerly Firmicutes) (18.08%), and Pseudomonadota (formerly Proteobacteria) (9.02%) as the dominant phyla in the gut microbiota of *A. pernyi* larvae. Previous studies have indicated that the dominance of Actinobacteria in the insect gut is a result of long-term co-evolution with their hosts. Actinobacteria in the insect gut assist the host in digesting food, defending against pathogenic infections, and providing nutritional resources, among other functions [22]. As early as 1946, Hungate demonstrated that *Micromonospora propionici* in the Termitidae gut aids the host in degrading cellulose [23]. *Rhodococcus rhodnii* provides essential vitamins to the insect host *Rhodnius prolixus*, ensuring normal growth and reproduction [24]. Bioactive compounds produced by *Pseudonocardia* in the gut of *Apterostigma dentigerum* can selectively inhibit the growth of the pathogen *Escovopsis* sp. [22]. Therefore, as revealed in this study, the abundant Actinobacteria identified in the larval gut of the *A. pernyi* during the 1–4 instars may also represent a potentially valuable microbial resource. This finding could provide insights for further research on the healthy rearing of the *A. pernyi*.

In contrast, Cyanobacteriota overwhelmingly dominated (90.67%) the gut microbiota of 5th-instar larvae, aligning with its high abundance detected in the midgut and midgut contents of this developmental stage [19,25]. A similar pattern was observed in other lepidopteran species: Cyanobacteriota constituted a significant proportion (>84.76%) of the gut microbiota in female *Bombyx mori* [26], and a high richness was also reported in *Orthosia songi* larvae [27]. Additionally, Cyanobacteria have been detected in the gut of *Mythimna separata* [28]. These phenomena may stem from insect feeding habits and methodological limitations: As organelles of endosymbiotic origin, chloroplast genomes retain 16S rRNA genes derived from Cyanobacteria. During non-specific amplification using 16S rRNA universal primers, the primers may detect not only target cyanobacterial genes but also chloroplast DNA released during the digestion process of phytophagous lepidopteran larvae. This constitutes an unavoidable challenge in current research on the gut microbiota of herbivorous insects, which could be addressed through innovative technological approaches and optimization of primer design in future studies.

In this study, Bacillota were present across all instar stages of *A. pernyi* larvae. Currently, *Bacillus*, as a major bacterial group in the gut capable of producing cellulases and proteases, has been widely utilized as a beneficial bacterium in animal husbandry. Previous studies have confirmed that certain strains of *Bacillus (e.g., Bacillus amyloliquefaciens* and *Bacillus subtilis*) can be incorporated into the diet of *A. pernyi* to enhance digestion, absorption, and feed conversion efficiency [7,29]. Additionally, empty-gut soft disease frequently occurs in *A. pernyi* rearing, which is caused by infection with *Streptococcus*. Research has demonstrated that *Bacillus amyloliquefaciens* can not only serve as a probiotic to reduce disease prevalence but also enhance the antibacterial capacity of the larvae through the production of secondary metabolites [30]. Therefore, further exploration of beneficial strains can be conducted based on the analysis and discussion of this study.

Furthermore, at the genus taxonomic level, this study identified *Dietzia*, unclassified_f__Nitriliruptoraceae, *Nesterenkonia*, and *Lactobacillus* as the predominant gut microbiota of *A.pernyi* larvae. Differential richness analysis revealed statistically significant dynamic changes in these microbial populations during larval development. Notably, *Dietzia* emerged as the dominant genus in 2nd–3rd-instar larvae. Members of this genus possess remarkable catabolic capabilities toward hydrophobic hydrocarbons and sulfonamide antibiotics, potentially facilitating host detoxification of recalcitrant environmental pollutants [31]. This symbiotic relationship may compensate for the underdeveloped metabolic detoxification systems in early instar larvae. A remarkable microbial succession was observed at the 4th-instar stage, with *Staphylococcus* becoming the predominant genus. While many staphylococcal species exist as commensals involved in nutrient cycling, this genus includes opportunistic pathogens such as *Staphylococcus aureus* that may cause infections in immunocompromised hosts.

Based on LefSe and significance testing, the study confirmed that the gut microbiota structure of *A. pernyi* larvae varies significantly across different developmental stages, with notable changes in microbial abundance as the larvae grow. At the 1st-instar stage, Pseudomonadaceae was the dominant microbiota. The 3rd-instar stage exhibited the highest gut microbiota diversity, with Actinobacteria predominating at the phylum level, at the class level, Acidimicrobia, Actinobacteria, and Gammaproteobacteria were prominent, and at the genus level, seven key microbiota genera, including *Alteribacter* and *Aquihabitans* were clustered. The 4th-instar stage was enriched with Propionibacteriales and Geodermatophilus. These dynamic changes in gut microbiota may hold significant biological implications for the host at different developmental stages, warranting further in-depth investigation.

In this study, alpha diversity analysis revealed that the gut microbial diversity of the larvae exhibited an initial increase followed by a decrease with larval development, reaching its lowest point at the 5th instar. The 5th-instar larvae showed the lowest ACE and Chao indices, as well as the lowest Shannon index and highest Simpson index, indicating the lowest richness and diversity in their gut microbiota. These findings are consistent with reported decreases in microbiota during late instars of *Spodoptera littoralis*, potentially associated with gut content clearance preceding pupation in final instar larvae [32].

Beta diversity analysis revealed significant compositional shifts in the gut microbiota across developmental instars of *A. pernyi*, mirroring documented structural variations during *Spodoptera frugiperda* ontogeny [33]. Such dynamic patterns may stem from instar-specific behavioral adaptations that differentially shape microbial colonization. Furthermore, insect gut microbiota is known to be modulated by multifactorial determinants including dietary intake, social interactions, and environmental microbial exposure. Comparative studies on polyphagous species (e.g., *Colletes gigas*) and eusocial insects (e.g., *Apis mellifera* and *Solenopsis invicta*) have similarly demonstrated stage-dependent microbial community restructuring [34,35].

Notably, this study establishes that even in phytophagous insects with simplified ecological niches and monophagous diets (exclusively consuming *Quercus* leaves), gut microbiota composition undergoes significant stage-specific reorganization. Proposed mechanisms include: (1) vertical microbial transmission via eggshell consumption in early instars, and (2) expanded environmental microbial recruitment in later instars due to increased feeding activity and spatial mobility [36,37]. However, the precise ontogenetic trajectory of microbiota assembly and its underlying regulatory mechanisms warrant further systematic investigation.

Analysis of KEGG functional prediction based on PICRUSt2 revealed that the gut microbiota of *A. pernyi* larvae may be extensively involved in key physiological processes, such as Amino acid metabolism, Carbohydrate metabolism, Energy metabolism, and Cofactor and vitamin metabolism. These findings suggest that the nutrients in the leaves consumed by the larvae may not fully meet their metabolic and developmental requirements, necessitating assistance from gut microbes. Previous studies have reported that gut bacteria in four lepidopteran species feeding on mulberry trees also function in Carbohydrate and amino acid metabolism [38]. For instance, *Stenotrophomonas* in the gut of *B.mori* larvae provides essential amino acids, significantly enhancing their adaptability, thereby increasing larval resistance to chlorpyrifos and reducing the toxicity of organophosphorus insecticides [38]. This confirms that gut microbiota can improve host ecological adaptability through nutritional supplementation. Further analysis of the differences in gut bacterial gene functions revealed that metabolic-related functional genes were generally higher and exhibited little variation from the 1st to 4th-instar larvae, whereas carbohydrate and amino acid metabolism-related genes were significantly lower in the 5th instar compared to other instars. In contrast, Energy metabolism and Cofactor and vitamin metabolism-related genes were markedly elevated. This may be related to the larval life cycle, as 5th-instar larvae cease feeding and prepare for pupation, leading to an overall reduction in metabolic activity while their mobility (e.g., crawling, silk spinning, and cocoon formation) increases, demanding higher energy expenditure. However, the PICRUSt2-based functional prediction used in this study cannot directly confirm the actual functions of these microbiota or their direct impact on host growth and development. Further validation through metagenomics or other experimental approaches is required.

## 5. Conclusions

Lepidopteran insects and gut microbiota exhibit complex interaction mechanisms and symbiotic relationships, making in-depth research on insect gut microbiota highly significant [14,39,40,41]. This study focuses on the entire developmental period of *A. pernyi* larvae. Using the Illumina Miseq high-throughput sequencing platform, we conducted 16S rRNA-based amplicon sequencing on laboratory-reared *A. pernyi* larvae, analyzing in detail the composition and diversity characteristics of their gut microbiota across all five instars (1st to 5th). Additionally, we explored the potential roles of the microbiota in the development of *A. pernyi* through functional prediction. In this study, a total of 18 phyla, 33 classes, 81 orders, 137 families, and 270 genera of gut microbiota were detected, a number significantly higher than the 10 phyla, 20 classes, 40 orders, 57 families, and 71 genera reported in previous studies from the intestinal tracts of fifth-instar *A. pernyi* larvae [19]. This fully reflects the significance and value of studying *A. pernyi* gut microbiota from a holistic and systemic perspective. This study systematically reveals the dynamic succession patterns and potential functions of gut microbiota in *A. pernyi* larvae across developmental instars, enriching the gut microbial resource library for lepidopteran insects. It lays an important foundation for understanding host-microbe interactions in lepidopteran insects. However, the specific molecular interaction mechanisms between the host and gut microbiota, how the microbiota collaborates with the host to adapt to complex and changing environmental stressors, and the precise physiological and ecological functions of key genera such as *Dietzia* and *Staphylococcus* observed in this study remain core scientific questions that require further exploration in the future.

## Figures and Tables

**Table 1:** Basic information on the high-throughput sequencing of the microbiota 16S rRNA.

Samples		Valid Reads	Average Length (bp)	Max Length (bp)	Min Length (bp)	Number of ASV
AP1	AP1_1	62,075	408	430	201	168
	AP1_2	69,332	417	431	224	120
	AP1_3	71,839	421	518	201	148
	Total	203,246	415	518	201	436
AP2	AP2_1	61,412	409	435	203	75
	AP2_2	68,912	420	433	212	121
	AP2_3	67,337	417	527	201	156
	Total	197,661	415	527	201	352
AP3	AP3_1	43,177	413	431	221	124
	AP3_2	68,972	412	431	219	120
	AP3_3	72,712	413	485	205	149
	Total	184,861	417	485	205	393
AP4	AP4_1	42,761	407	529	203	166
	AP4_2	65,513	410	437	208	189
	AP4_3	44703	427	498	221	44
	Total	152,977	415	529	203	399
AP5	AP5_1	62,320	406	430	262	23
	AP5_2	56,434	407	430	225	96
	AP5_3	65,707	406	527	267	41
	Total	184,461	406	527	225	160

Note: AP (1-1, 1-2, 1-3),1st-instar larvae; AP (2-1, 2-2, 2-3), 2nd-instar larvae; AP (3-1, 3-2, 3-3), 3rd-instar larvae; AP (4-1, 4-2, 4-3), 4th-instar larvae; AP (5-1, 5-2, 5-3), 5th-instar larvae.

**Table 2:** Statistical data of gut microbiota Alpha diversity of *A. pernyi* larvae across different instars.

Samples	Richness		Diversity	
	ACE	Chao	Shannon	Simpson
AP1	145.32 ± 24.57	145.33 ± 24.70	2.66 ± 0.63	0.22 ± 0.17
AP2	117.91 ± 41.19	117.75 ± 41.00	2.744 ± 0.40	0.16 ± 0.06
AP3	131.11 ± 15.76	131.00 ± 15.72	2.79 ± 0.17	0.16 ± 0.26
AP4	133.00 ± 77.93	133.00 ± 77.93	2.01 ± 0.96	0.37 ± 0.22
AP5	53.07 ± 38.40	53.00 ± 38.43	0.63 ± 0.76	0.82 ± 0.21

Note: Data were mean ± SE. AP1, 1st-instar larvae; AP2, 2nd-instar larvae; AP3, 3rd-instar larvae; AP4, 4th-instar larvae; AP5, 5th-instar larvae.

## Data Availability

All data presented in this study are included in this article. For further inquiries, please contact the first author.
